# Increased γ-Secretase Activity in Idiopathic Normal Pressure Hydrocephalus Patients with β-Amyloid Pathology

**DOI:** 10.1371/journal.pone.0093717

**Published:** 2014-04-03

**Authors:** Tiina Laiterä, Timo Sarajärvi, Annakaisa Haapasalo, Lakshman Puli, Tarja Kauppinen, Petra Mäkinen, Tuomas Rauramaa, Heikki Tanila, Juha E. Jääskeläinen, Irina Alafuzoff, Hilkka Soininen, Ville Leinonen, Mikko Hiltunen

**Affiliations:** 1 Institute of Clinical Medicine - Neurosurgery, University of Eastern Finland, Kuopio, Finland; 2 Neurosurgery of NeuroCenter, Kuopio University Hospital, Kuopio, Finland; 3 Institute of Clinical Medicine - Neurology, University of Eastern Finland, Kuopio, Finland; 4 A.I. Virtanen Institute, University of Eastern Finland, Kuopio, Finland; 5 Institute of Clinical Medicine - Pathology, University of Eastern Finland, Kuopio, Finland; 6 Department of Pathology, Kuopio University Hospital, Kuopio, Finland; 7 Department of Immunology, Genetics and Pathology, Uppsala University, Uppsala, Sweden; 8 Department of Neurology, Kuopio University Hospital, Kuopio, Finland; Torrey Pines Institute for Molecular Studies, United States of America

## Abstract

The potential similarity between the brain pathology of idiopathic normal pressure hydrocephalus (iNPH) and Alzheimer disease (AD) is intriguing and thus further studies focusing on the underlying molecular mechanisms may offer valuable information for differential diagnostics and the development of treatments for iNPH. Here, we investigated β- and γ-secretase activities in relation to amyloid-β (Aβ) pathology in the brain tissue samples collected from iNPH and AD patients. β- and γ-secretase activities were measured from the frontal cortical biopsies of 26 patients with suspected iNPH as well as post-mortem tissue samples from the inferior temporal cortex of 74 AD patients and eight subjects without neurofibrillary pathology. In iNPH samples with detectable Aβ plaques, γ-secretase activity was significantly increased (∼1.6-fold) when compared to iNPH samples without Aβ plaques (*p* = 0.009). In the AD samples, statistically significant differences in the γ-secretase activity were not observed with respect to disease severity (mild, moderate and severe AD according to neurofibrillary pathology). Conversely, β-secretase activity was unaltered in iNPH samples with or without Aβ plaques, while it was significantly increased in relation to disease severity in the AD patients. These results show for the first time increased γ-secretase but not β-secretase activity in the biopsy samples from the frontal cortex of iNPH patients with AD-like Aβ pathology. Conversely, the opposite was observed in these secretase activities in AD patients with respect to neurofibrillary pathology. Despite the resemblances in the Aβ pathology, iNPH and AD patients appear to have marked differences in the cellular mechanisms responsible for the production of Aβ.

## Introduction

The normal pressure hydrocephalus (NPH), which typically presents symptoms including deteriorated gait, impaired cognition and urinary incontinence with enlarged ventricles and normal or slightly elevated cerebrospinal fluid (CSF) pressure is a potentially dementing condition [1], [2]. NPH can result from an earlier event, such as infection or trauma, or as idiopathic (iNPH) without any distinctive external cause [2]. Diagnostics of iNPH are hindered by the lack of knowledge of the underlying molecular causes and by the potential similarity in brain pathology with Alzheimer disease (AD), including the accumulation of amyloid-β (Aβ) and/or tau proteins [3]. Importantly, AD is along with vascular dementia the most frequent differential diagnosis to iNPH [4]. This, together with the frequency of AD-related changes in iNPH, has raised the question whether iNPH and AD share similar pathogenic processes [3].

Two important aspartyl proteases involved in amyloid precursor protein (APP) processing and amyloid-β (Aβ) peptide formation are the β- (BACE1) and γ-secretases. It is well-established that AD patients show approximately a 30% increase in the β-secretase levels and activity as compared to the age-matched, non-demented subjects [5]–[7]. Conversely, no differences in γ-secretase activity have been detected between AD patients and non-demented subjects, although some qualitative alterations are suggested to occur [8], [9]. Here, we set our goal to investigate whether alterations in β- and γ-secretase activities in relation to Aβ pathology are observed in the biopsy samples obtained from the frontal cortex of the iNPH patients. Furthermore, β- and γ-secretase activities from the inferior temporal cortex of AD patients were assessed in comparison.

## Materials and Methods

### Biopsy Cohort

Neurosurgery of Kuopio University Hospital (KUH) has provided the defined study population in Eastern Finland. At the end of the year 2012, the Kuopio NPH Registry (www.uef.fi/nph) consisted of 733 patients evaluated for assumed NPH fulfilling the following criteria: 1) one to three symptoms peculiar to NPH: impaired cognition, gait or urinary continence; 2) enlarged brain ventricles compared to the size of the sulci of cerebral convexities in computed tomography (CT) or magnetic resonance imaging (MRI); 3) brain biopsy available [4]. For this study, available fresh frozen right frontal brain cortex biopsies collected from 26 patients (mean age 76.5± SD 6.1; range 63.5 to 87.5 years; 42.3% female) with suspected iNPH were employed (Table 1). The biopsy procedure has been described in detail previously [4]. In outline, a right frontal 12 mm burr hole was made approximately three cm laterally from the midline and close to the coronal suture of the skull under local anesthesia and sedation (ICP monitoring) or general anesthesia (shunt procedure). Prior to the insertion of an intraventricular catheter for 24-hour ICP monitoring or alternatively prior to insertion of intraventricular shunt, one to three cylindrical cortical brain biopsies of two mm in diameter and three to seven mm in length were obtained with biopsy forceps or by standard 14G biopsy needle (Temno).

**Table 1:** Demographic characteristics of patients with suspected iNPH.

	n	Age, y, mean ± SD	Female, n (%)	MMSE, mean ± SD	Shunt/response (%)	Symptoms			Immunoreactivity	
						Gait difficulty	Impaired cognition	Urinary incontinence	NFT -	NFT +
**All patients**	26	76.5±6.1	11 (42.3)	20.0±6.3	19/13 (68.4)	26	23	18	20	6
**Aβ −**	8	74.6±5.9	3 (37.4)	22.0±5.5	5/2 (40.0)	8	7	5	8	0
**Aβ +**	18	77.4±6.1	8 (44.4)	19.1±6.5	14/11 (78.6)	18	16	13	12	6

Abbreviations: Aβ − = no β-amyloid in the biopsy; Aβ+ =  β-amyloid in the biopsy; MMSE = Mini-Mental State Examination; NFT − = no tau neurofibrillary tangles in the biopsy; NFT+ =  tau neurofibrillary tangles in the biopsy.

### Neuropathological Cohort

Human post-mortem brain samples were obtained from Kuopio University Hospital (Kuopio Brain Bank; http://www.uef.fi/fi/neuro/alzheimer). This set included inferior temporal lobe samples from 82 older individuals investigated within memory clinic research projects and later autopsied and evaluated for AD pathology. The CERAD criteria were used for the diagnosis of AD [10]. AD patients were subdivided in three severity groups; mild (n = 35), moderate (n = 12) and severe (n = 27) according to Braak staging (1–2 = mild, 3–4 = moderate, 5–6 = severe) (Table 2) [11]. Furthermore, eight subjects without AD-related neurofibrillary pathology ( =  Braak 0 group) were used for the comparison purposes in the subsequent analyses (Table 2).

**Table 2:** Demographic and clinical characteristics of the AD patients and Braak 0 subjects.

Severity (number of subjects)^a^	Braak staging	Number of subjects/stage	Females, n (%)	Age at death, y,mean ± SD^b^	PMD, h,mean ± SD^c^	MMSE Score, mean ± SD^d^
**Braak 0** (n = 8)	0	8	3 (38)	68±16	13±15	22±10
**MILD** (n = 35)	1	14	7 (50)	83±6.4	18±23	20±6.4
	2	21	16 (76)			
**MODERATE** (n = 12)	3	5	4 (80)	84±7.6	16±17	15±6.4
	4	7	6 (86)			
**SEVERE** (n = 27)	5	16	14 (88)	82±7.4	6.5±4.5	16±6.4
	6	11	11 (100)			

a) Classification to mild, moderate and severe according to Braak staging; 1–2 = mild, 3–4 = moderate, 5–6 = severe. Braak stage 0 =  subjects without neurofibrillary pathology.

b) There were no significant differences in the age of death between mild, moderate, and severe groups in the AD cohort.

c) Post-mortem delay; significant difference in the post-mortem delay between mild and severe groups (*p* = 0.013).

d) MMSE = Mini-Mental State Examination.

### Ethics Statement

Written consent was obtained from all subjects. The study was approved by the Kuopio University Hospital ethical committee, the Finnish National Supervisory Authority, University of Eastern Finland, and the Finnish Ministry of Social Affair and Health.

### Processing of the Tissue Samples

Frozen samples from both iNPH and AD patients were mechanically homogenized in 400 μl of 1× buffer B (20 mM Hepes pH 7.5, 150 mM KCl, 2 mM EGTA) with protease and phosphatase inhibitors (1∶100; Thermo Scientific) in an ice bath. Protein aliquots were then ultracentrifuged (100000×*g*, 50.4 Ti rotor; Beckman) for two hours at 4°C, and the supernatant ( =  soluble fraction) was collected for soluble Aβ x-42 peptide measurements. Subsequently, the remaining pellet was re-suspended in the 200 μl of guanidine buffer (5 M guanidine-HCl/50 mM Tris-HCl, pH 8.0), incubated for 2 hours at room temperature on a shaker, and diluted 1∶50 in BSAT-DPBS (5% BSA/0.03% Tween-20 in DPBS, pH 9.0) containing protease and phosphatase inhibitors. The suspension was centrifuged for 20 minutes at 15700×*g* and the supernatant was collected for β- and γ-secretase enzyme activity assays.

### Soluble Aβ x-42 Measurements

Soluble Aβ x-42 (Aβ42) levels were measured from the soluble fraction. The soluble Aβ42 levels were determined using a monoclonal and HRP-conjugated antibody-based Human/Rat *β* Amyloid 42 (High-Sensitive; 290-62601) ELISA Kit (Wako). After a 30-minute incubation at room temperature, the reaction was terminated and the absorbance was measured at 450 nm with an ELISA microplate reader (BioRad). Aβ42 levels were normalized to total protein levels within each sample.

### β- and γ-secretase Activity Assays

β-Secretase (Cat # K360-100, BioVision, CA, USA) activity was measured from the tissue homogenates according to the manufacturer’s instructions. Briefly, membrane protein fractions at the final concentration of 0.01 μg/μl were incubated at 37°C for 1 hour with the β-secretase-specific substrate peptides conjugated to fluorescent reporter molecules EDANS and DABCYL. Subsequently, the emitted light 510 nm was detected on a fluorescence microplate reader (Wallac) after EDANS excitation at 355 nm. γ-secretase activity was measured from the tissue homogenates as previously described [12]. In brief, solubilized membrane protein fractions at the final concentrations of 0.20 μg/μl and 0.13 μg/μl for AD and iNPH samples, respectively, were incubated at 37°C overnight in 150 μl of assay buffer containing 50 mM Tris-HCl, pH 6.8, 2 mM EDTA, 0.7% CHAPSO (w/v), and 8 μM fluorogenic γ-secretase substrate (NMA-GGVVIATVK(DNP)- D R D R D R-NH 2, Cat # 565764, Calbiochem). After incubation, samples were centrifuged at 15700×*g* for 10 min and transferred to a 96-well plate. Fluorescence was measured using a plate reader (Fluorstar Galaxy) with an excitation wavelength of 355 nm and an emission wavelength of 440 nm. The background fluorescence from substrate samples was subtracted in the final analysis. β- (β-Secretase Inhibitor III, GL189, Cat # 565780, Calbiochem; 150 μM/reaction) and γ- (L-685,458; Cat # L1790, Sigma-Aldrich; 100 μM/reaction) secretase inhibitors were used in a subset of iNPH and AD samples to validate the specificity of the β- and γ-secretase activities (Figure S1).

### Histology and Immunohistochemistry

Part of the iNPH biopsy samples were fixed in buffered formalin overnight and embedded in paraffin. The 26 paraffin-embedded biopsy samples were sectioned (7 μm) and stained with hematoxylin-eosin, and immunostained with monoclonal antibodies directed to Aβ (6F/3D, M0872; Dako; dilution 1∶100; pre-treatment 80% formic acid 1 hour) and phosphorylated tau protein (p-tau) (AT8, ^3^Br-3; Innogenetics; dilution 1∶30) [4]. Aβ was semi-quantified by counting plaques in the biopsy under a light microscope and dividing the total number of plaques by the area of the gray matter (mm^2^). Cellular or neuritic immunoreactivity for p-tau was evaluated by light microscopy in all samples and was graded as present or absent by a neuropathologist [13]. Aβ was also quantified more precisely by a method described earlier [13]. Briefly, representative high-resolution images consisting of the cortical regions of interest were acquired at 2X magnification (Plan N2X/0.06) using an upright Olympus optical microscope (OLYMPUS BX40) with Olympus optical DP50 camera. A flatfield image was also acquired under similar settings for correcting uneven illumination. On the grey-scaled images, cortical regions of interest were outlined and selected using Lasso tools. Images were then thresholded to segregate plaques from the background. The number of pixels counted within selections, after calibration, gave corresponding areas in mm^2^. Percentage of cortical area covered with stained antibody against Aβ was reported for the biopsy samples.

### Statistical Analyses

Statistical analyses were performed using the SPSS program (version 19.0). One-way ANOVA with a post-hoc test (LSD) was used for statistical analyses of biochemical data. Comparisons between groups were made using independent samples t-test and non-parametric Mann-Whitney U-test. Correlations were determined using Pearson’s correlation coefficient. Values are indicated as mean ± SE. The level of statistical significance was set to *p*<0.05.

## Results

### Soluble Aβ42 Levels and the Relative Immunohistochemical Quantification of Aβ Correlate with the Visual Aβ Plaque Density and the Presence of NFTs in the Frontal Cortex of iNPH Patients

**Figure 1:**
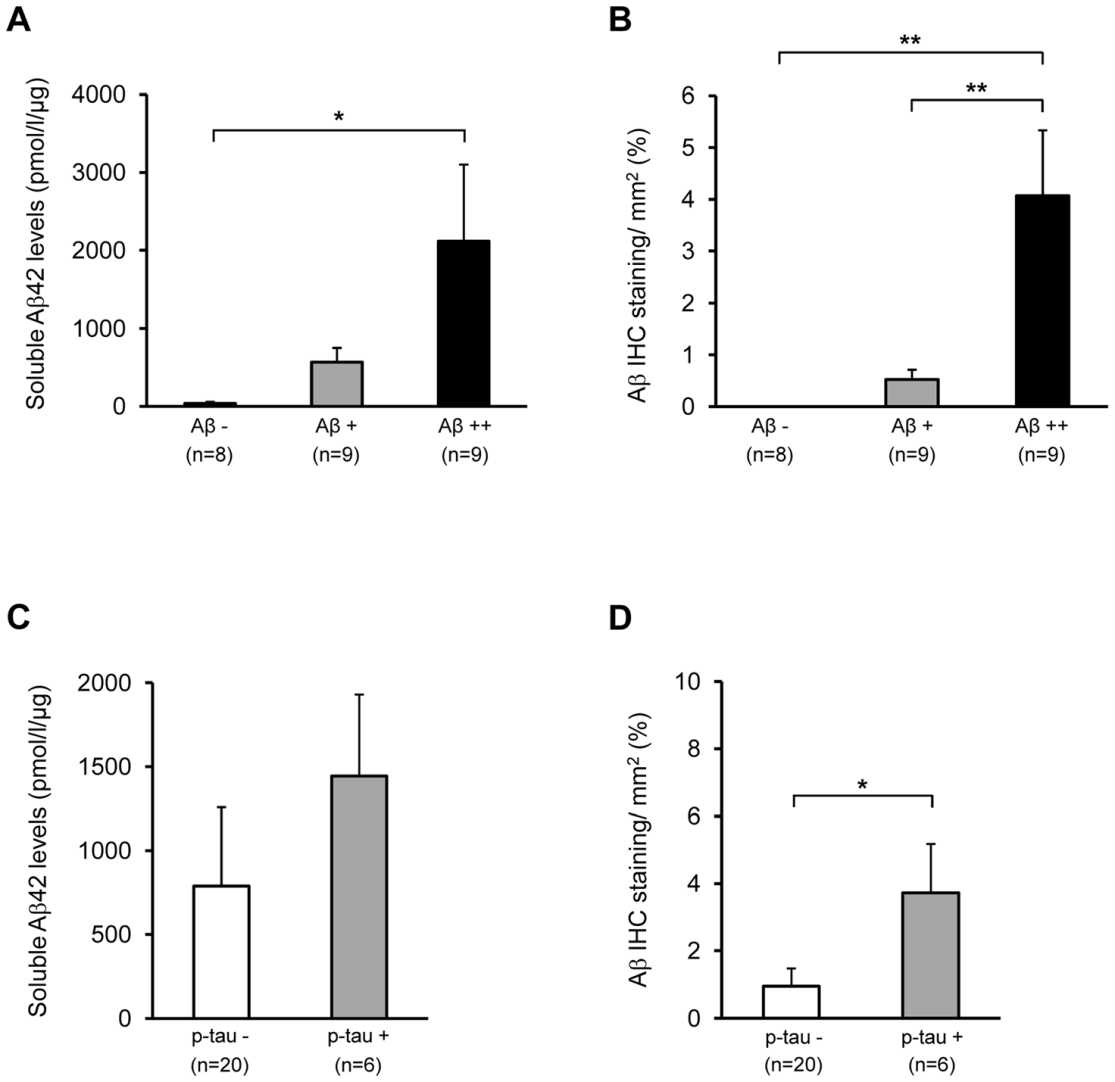
Soluble Aβ42 levels and relative Aβ staining are increased in the frontal cortex of iNPH patients with Aβ pathology. (A, B) Brain samples were immunostained against Aβ: Aβ − = no plaques, Aβ+ =  less than 100 plaques and Aβ++ =  more than 100 plaques per section (visual plaque count). A significant increase in the soluble Aβ42 levels is observed between Aβ - and Aβ++ samples. In addition, a significant increase in the relative Aβ intensity [immunohistochemical Aβ−staining/mm2 (%)] is observed between both Aβ - and Aβ ++, and Aβ + and Aβ ++ samples. (C, D) Brain samples were immunostained using AT8 antibody against NFTs: NFT− = no NFTs obsrved, NFT+ =  NFTs observed. Relative Aβ levels were significantly increased in NFT+samples compared to NFT -. Data are shown as mean ± SE, **p<0.01, *p<0.05.

iNPH patient samples (n = 26) acquired from the frontal cortex were divided into three groups based on visual analysis performed by neuropathologists [13]. Samples with no visually detectable Aβ plaques in the histological section were marked as Aβ -, while the samples with less than 100 plaques per section were marked as Aβ+ and those with more than 100 plaques as Aβ ++. Consequently, soluble Aβ42 levels as well as Aβ− staining values obtained from the immunohistochemical quantification analysis (the percentage of cortical area stained with an antibody against Aβ) were compared to the visually detectable Aβ plaque counts (Figure 1A and 1B). Both soluble Aβ42 levels and the relative Aβ Aβ− staining values were increased in a stepwise manner in relation to the Aβ plaque counts (Aβ −, Aβ+ and Aβ ++). The age of the iNPH patients at the biopsy did not significantly vary between Aβ−negative and Aβ−positive iNPH sample groups (*p* = 0.28) (Table 1). Furthermore, the age of the iNPH patients at the biopsy did not significantly correlate with soluble Aβ42 levels (*r* = 0.201, *p* = 0.33) or with the relative Aβ−staining values (*r* = 0.414, *p* = 0.09). In addition, immunohistochemical staining against the tau neurofibrillary tangles (NFTs) was performed from the same sample sections and compared to the soluble Aβ42 levels and relative Aβ−staining values (Figure 1C and 1D). Six iNPH patients were found positive for NFT-staining and a significant increase in Aβ−staining was observed in NFT-positive iNPH patients as compared to the NFT-negative patients. Soluble Aβ42 levels did not significantly differ with respect to the NFT status.

### γ-secretase, but not β-secretase Activity is Increased in the Frontal Cortex of iNPH Patients with AD-like Aβ Pathology

**Figure 2:**
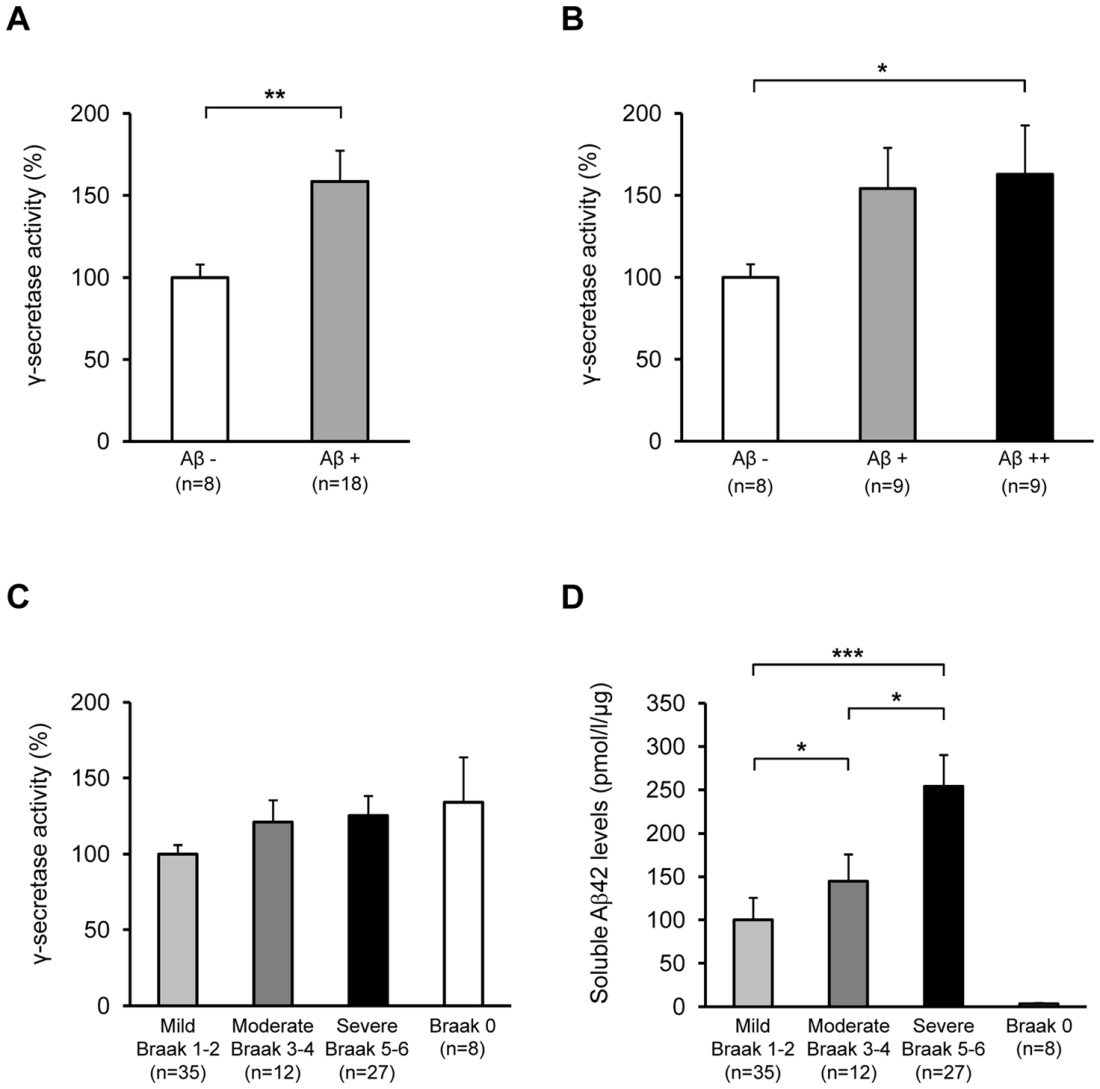
γ-secretase activity is increased in the frontal cortex of iNPH patients with Aβ−pathology. (A) Visual plaque count from immunostained brain sections: Aβ − = no plaques, Aβ+ =  plaques observed. Significant increase in γ-secretase activity is observed in Aβ+samples compared to Aβ - samples. (B) Aβ − = no plaques, Aβ+ =  less than 100 plaques and Aβ++ =  more than 100 plaques per section. Increase in γ-secretase activity (not significant) can be observed along with increased Aβ−status. (C, D) Significant increase is observed in soluble Aβ42 levels but not in γ-secretase activity in the inferior temporal cortex of AD patients in relation to disease pathology (Braak 0 =  subjects without neurofibrillary pathology, 1–2 = mild, 3–4 = moderate, and 5–6 = severe). Mild AD group was normalized to 100%. Soluble Aβ42 levels in Braak 0 group were significantly decreased when compared to mild, moderate and severe groups (*p*<0.05). Data are shown as mean ± SE, ***p<0.001, **p<0.01, *p<0.05.

**Figure 3:**
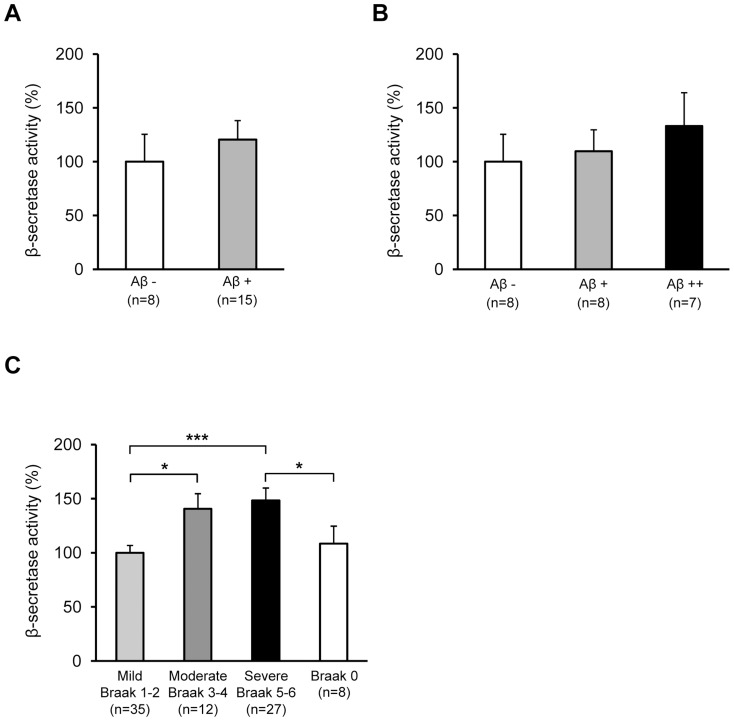
β-secretase activity is unaltered in the frontal cortex of iNPH patients with Aβ−pathology. (A, B) Visual plaque count from immunostained brain sections. (A) Aβ − = no plaques, Aβ+ =  plaques observed. (B) Aβ − = no plaques, Aβ+ =  less than 100 plaques and Aβ++ =  more than 100 plaques per section. No significant alteration in β-secretase activity is observed between different iNPH sample groups. (C) β-secretase activity in AD is significantly increased in the inferior temporal cortex of AD patients in relation to disease pathology (Braak 0 =  subjects without neurofibrillary pathology, 1–2 = mild, 3–4 = moderate, and 5–6 = severe). Mild AD group was normalized to 100%. Data are shown as mean ± SE, ***p<0.001, *p<0.05.

**Figure 4:**
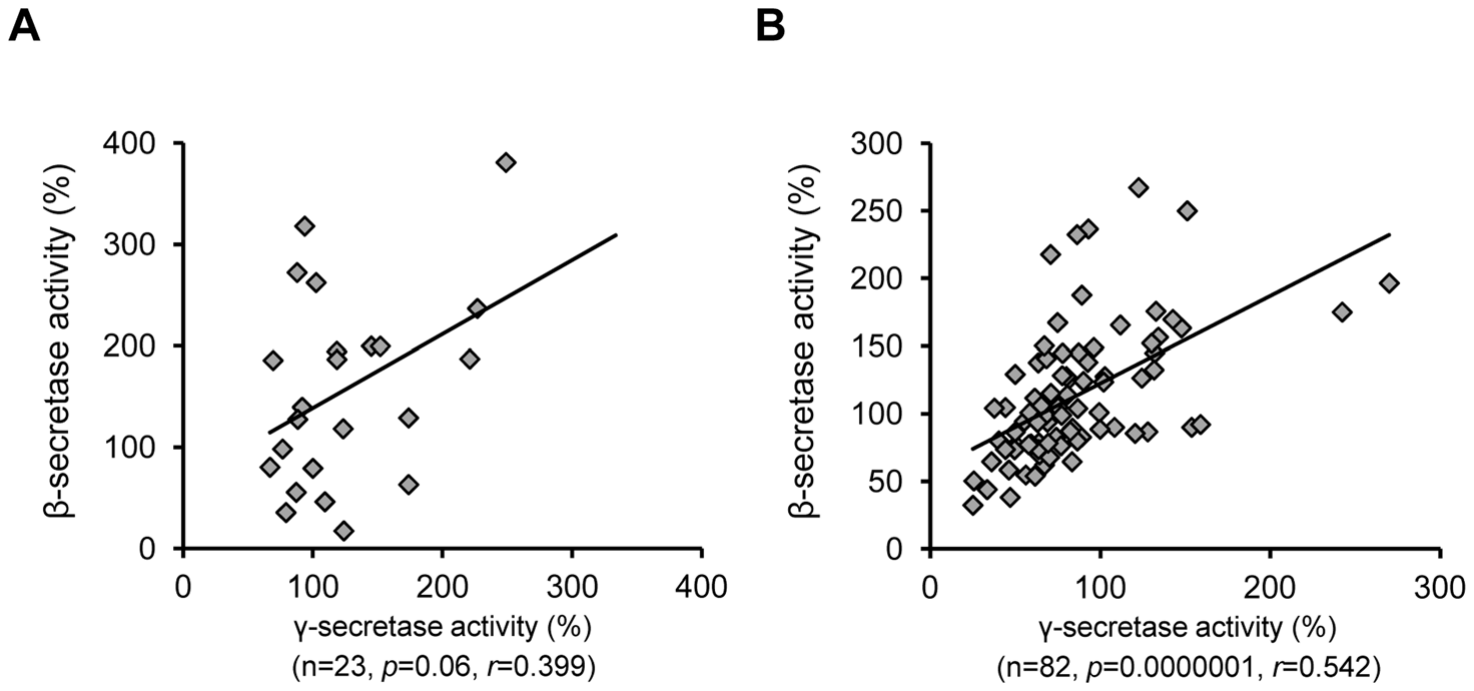
β- and γ-secretase activities show positive correlation among the AD patients. (A, B) Pearson’s correlation between relative β- and γ-secretase activities measured in the brain samples iNPH (A) and AD (B) patients. Statistically significant positive correlation is observed in the AD sample cohort, but not in the iNPH cohort.

Before assessing β- and γ-secretase activities in the iNPH and AD tissue samples, both activity assays were validated using β- and γ-secretase-specific inhibitors. Activity measurements of the inhibitor-treated biopsy and post-mortem lysates revealed significant decrease in the β- and γ-secretase activity (Figure S1). Consequently, relative γ-secretase activity was found to be significantly higher in the iNPH samples with Aβ−plaques (Aβ+and Aβ++ samples) as compared to the iNPH samples without detectable Aβ−plaques (*p = *0.009; Figure 2A and 2B). Conversely, no significant differences in the relative γ-secretase activity were observed in the inferior temporal cortical samples of AD patients with respect to disease severity (divided according to the Braak staging into mild, moderate and severe groups [11]) (Figure 2C). Moreover, comparison between different severity groups and subjects without neurofibrillary pathology ( =  Braak 0 group) did not reveal significant changes in the relative γ-secretase activity. Soluble Aβ42 levels were in turn increased significantly in relation to disease severity, while the soluble Aβ42 levels were robustly lower in the Braak 0 group as compared to mild, moderate and severe groups (Figure 2D). On the contrary to the increased γ-secretase activity, β-secretase activity was not significantly altered in the iNPH samples between different Aβ groups (Figure 3A and 3B), while a significant difference was observed in relation to AD severity in the AD cohort (*p = *0.0003 between mild and severe and *p* = 0.02 between mild and moderate; Figure 3C). Also, β-secretase activity was significantly increased in the severe group as compared to the Braak 0 group. Activity of β- or γ-secretase did not significantly correlate with the relative Aβ−staining values among the iNPH samples (*r* = −0.200, p = 0.47 and *r* = −0.101, *p* = 0.69, respectively). Also, β-secretase and γ-secretase activity did not significantly correlate in the iNPH sample set (*r = *0.399, *p = *0.06; Figure 4A), while a robust correlation between the activity of these two secretases was noticed in the AD sample set (*r = *0.542, *p = *0.0000001; Figure 4B). Activity of β- or γ-secretase did not significantly correlate with the post-mortem delay (*r* = −0.177, *p* = 0.10 and *r* = −0.101, *p* = 0.36, respectively) or with the age of death (*r* = 0.142, *p* = 0.20 and *r* = 0.096, *p* = 0.38, respectively) in the AD cohort. Moreover, activity of β- or γ-secretase did not significantly correlate with the age of iNPH patients at the biopsy (*r* = −0.183 and *p* = 0.59 and *r* = −0.110 and *p* = 0.40, respectively).

## Discussion

The present study was performed in order to investigate whether alterations in β- and γ-secretase activities in relation to the Aβ status could be observed in the biopsy samples collected from the frontal cortex of the iNPH patients. This originated from the idea that the marked Aβ pathology observed in a subset of iNPH patients could result at least in part from the enhanced production of Aβ. For comparison, β- and γ-secretase activities were also measured from the inferior temporal cortex of AD patients and subjects without neurofibrillary pathology. Before assessing the secretase activities according to the Aβ pathology, however, we wanted to validate whether the visually detected Aβ plaque counts correlated with more quantitative measures, such as soluble Aβ42 levels and relative immunohistochemical Aβ staining, in the sample set. According to our results, both soluble Aβ42 levels and relative Aβ staining revealed a significant difference between Aβ−negative and Aβ−positive iNPH sample groups, thus validating the sample grouping based on the visual Aβ plaque counts.

The most intriguing finding of the study was the relationship between γ-secretase activity and the Aβ status in the iNPH patient samples. iNPH samples with detectable Aβ plaques showed augmented γ-secretase activity as compared to iNPH samples without Aβ plaques. Furthermore, the increase in the γ-secretase activity appeared to be dependent on the amount of Aβ plaques, indicating an expected link between increased γ-secretase activity and the Aβ production. On the other hand, correlation analysis between γ-secretase activity and relative Aβ−staining values did not reveal significant correlation in the iNPH patients. The lack of significant correlation between these measures may reflect the complex equilibrium between the production and clearance of Aβ in the brain and/or relatively small sample size of iNPH patients. It is well-established that the alterations in the degradation and clearance status of Aβ play an important role in AD pathogenesis [14]. Similarly, it is anticipated that the Aβ pathology observed in the iNPH patients may be linked to the simultaneous alterations in both production- and degradation/clearance-related events, which in turn could complicate the assessment of direct relationship between γ-secretase activity and Aβ pathology. Interestingly, γ-secretase activity was not significantly affected in the AD samples with respect to disease severity or when different AD patient groups were compared to the subjects without neurofibrillary pathology, which suggest that the molecular mechanisms underlying the production of Aβ may be different between iNPH and AD. In line with these results, a recent study performed on patients with mild cognitive impairment (MCI) and AD showed no increase in overall γ-secretase activity in the brain tissue samples as compared to non-demented control samples [8]. On the contrary to the γ-secretase activity, β-secretase activity did not show alterations between iNPH samples with or without Aβ plaques. However, β-secretase activity was increased in relation to mild, moderate and severe groups in the AD cohort as shown previously [15] and when the severe AD patients were compared to the subjects without neurofibrillary pathology. [14].

Recent studies have implied an up-regulation of γ-secretase activity under hypoxic conditions and oxidative stress, which are considered to be both risk factors and clinical features of AD [16]–[18]. Also iNPH symptoms are suggested to be caused by impaired blood flow in the brain tissue surrounding the enlarged ventricles. Thus, ischemic and hypoxic stress conditions may have an effect on the neuropathology [19]. These stress conditions have been shown to affect the expression of presenilin-1 and −2, which are the catalytic subunits of the γ-secretase complex [20], [21]. Presenilin-1 appears to be needed for the normal induction of hypoxia-inducible factor 1α (HIF-1α) [21], [22]. Conversely, it has been shown that HIF-1α binds to the promoter of another γ-secretase complex component, the anterior pharynx-defective-1 (APH-1) [23], to up-regulate its expression. This in turn increases the activity of γ-secretase and the cleavage of APP [18]. The exact role of APH-1 in γ-secretase function is not completely clear, but according to these results with HIF-1α, it may have a direct effect on the γ-secretase activity independently of the other γ-secretase subunits.

β-secretase is also a stress-related protease [24], whose levels and activity are increased for example in hypoxia [25] and ischemia [26]. In the previous studies, it has been shown that AD patients have an approximately 30–50% increase in β-secretase activity as compared to age-matched non-demented subjects [5]–[7]. Increase in β-secretase activity has also been shown to directly link to increased production of Aβ40 and Aβ42 both *in vitro* and *in vivo* [27]. Since stress conditions related to iNPH and AD, such as ischemia and hypoxia, appear to be equally important for the increase in both β- and γ-secretase activities, it is somewhat puzzling that similar effects on the activity of these secretases cannot be observed in both iNPH and AD brain. Yet the disturbances in the brain metabolism might be less dramatic in iNPH than in AD, where impaired glucose metabolism is also considered as a common hallmark in addition to hypoxia and ischemia [16]–[18], [28].

Although it appears that the underlying molecular mechanisms related to Aβ production are different in iNPH and AD patients in terms of β- and γ-secretase activities, one should remember that there are potential confounding factors, which may have affected the observed outcome. Importantly, iNPH and AD samples originate from the different brain regions, which again could explain the observed differences in β- and γ-secretase activities. The rationale behind choosing these particular brain regions for each cohort was to investigate those brain regions known to be strongly affected in each disease: The frontal cortex exhibiting Aβ and NFT pathology in iNPH and the temporal cortex showing neurofibrillary and Aβ pathology in AD. Moreover, since the temporal cortex is more severely affected in AD than the frontal cortex according to the neuropathological changes, it is not likely that the γ-secretase activity would be augmented in the frontal cortex as this would also be expected to potentiate the Aβ pathology in that area. In addition, there may be alterations in the AD brain tissue due to different causes of death and post-mortem delay. Although there were no differences in the age of death between mild, moderate, and severe groups in the AD cohort, a significant difference was observed in the post-mortem delay between mild and severe groups in AD cohort. Then again, β- or γ-secretase activity did not significantly correlate with the post-mortem delay or with the age of death in the AD cohort, arguing against post-mortem- or age-related changes in the β- or γ-secretase activity in the temporal cortex. However, due to the potential AD-related events, such as alterations in the brain metabolism preceding the death, we cannot completely rule out the possibility that these confounding factors may have affected the biochemical outcome measures in the post-mortem samples relative to biopsy samples obtained from the living iNPH patients. Furthermore, both γ- and β-secretase activities have been shown to increase in the hypoxic and ischemic conditions [16]–[18], [24]–[26], and in the present study, the activity of these secretases correlated significantly with each other in the AD cohort. Thus, it is reasonable to conclude that the results obtained from the living iNPH patients may be pathophysiologically more relevant than those obtained from the post-mortem tissue samples, emphasizing further the value of findings related to the increased γ-secretase activity in the Aβ−positive iNPH patients. It should also be emphasized that other post-mortem-related events, such as long post-mortem delay, may have affected the reliability of some of the biochemical analyses. However, the fact that secretase activities and the post-mortem delay did not significantly correlate suggests that this is not likely. Finally, we did not observe correlation between the age of iNPH patients at the biopsy and β- or γ-secretase activity, suggesting that the observed increase in γ-secretase activity in the Aβ−positive group is mechanistically linked to other factors beyond the age of iNPH patients.

In conclusion, our results show for the first time the difference between iNPH and AD brain samples in terms of γ- and β-secretase activities. On the contrary to AD, iNPH biopsies showed an increase in γ-secretase activity in relation to the Aβ pathology, while β-secretase activity was unaltered. The opposite was true in the AD samples. Thus, it is intriguing that these crucial cleavage steps in the production of Aβ are differently altered in these two diseases, which show partially similar neuropathological phenotype. Importantly, our study suggests that the increased γ-secretase activity may play a key role in iNPH disease pathogenesis. There have been several attempts to develop γ-secretase inhibition-based therapies for the treatment of AD. However, γ-secretase complex shows broad activity towards various other substrates beyond APP [29], such as Notch [30]. Thus, the overt inhibition of γ-secretase activity may be harmful for the normal cellular functions, which could possibly explain why the clinical trials focusing on γ-secretase inhibition have so far failed in AD [31]. Nevertheless, it is tempting to speculate that restoring the normal γ-secretase activity in iNPH patients could offer a potential therapeutic approach to prevent AD-like Aβ pathology in these patients.

## Supporting Information

Figure S1 **Validation assays for β- and γ-secretase activity.** β- and γ-secretase inhibitors significantly decreased β- and γ-secretase activities (A) in the NPH tissue samples extracted from the frontal cortex and (B) in the post-mortem tissue samples extracted from the temporal cortex. Untreated sample was normalized to 100% in each case. Data are shown as mean ± SE, *p<0.05, n = 3–5.
(TIF)
