# Supplementary figures and images for: Increased γ-Secretase Activity in Idiopathic Normal Pressure Hydrocephalus Patients with β-Amyloid Pathology
Source: PLoS One. 2014 Apr 3;9(4):e93717. doi: 10.1371/journal.pone.0093717 (PMC3974803; doi:10.1371/journal.pone.0093717)

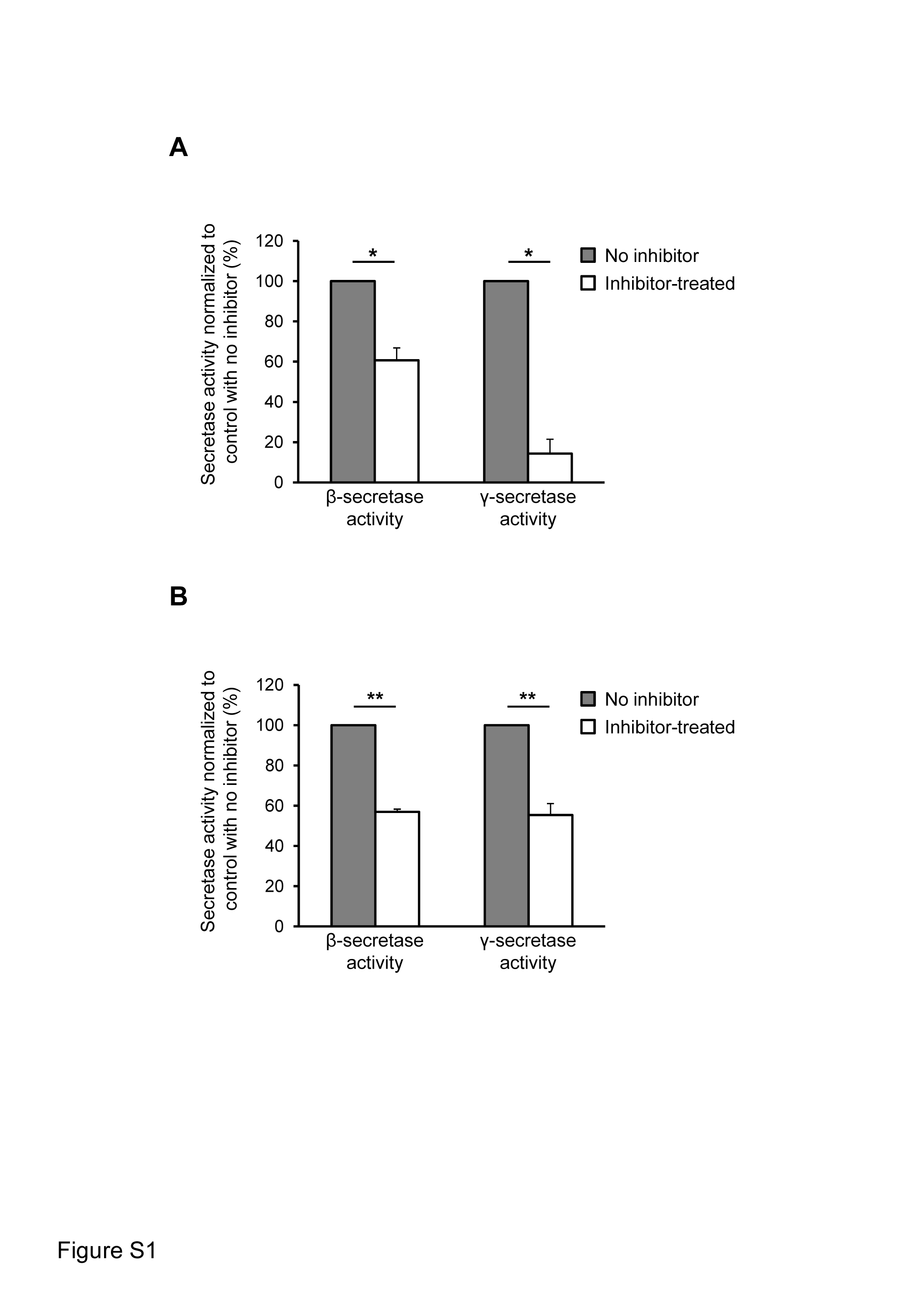

Supplement: Figure S1 — Validation assays for β- and γ-secretase activity. β- and γ-secretase inhibitors significantly decreased β- and γ-secretase activities (A) in the NPH tissue samples extracted from the frontal cortex and (B) in the post-mortem tissue samples extracted from the temporal cortex. Untreated sample was normalized to 100% in each case. Data are shown as mean ± SE, *p<0.05, n = 3–5. (TIF) [file pone.0093717.s001.tif]
